# Supplementary material for: The Human Cytomegalovirus β2.7 Long Non-Coding RNA Prevents Induction of Reactive Oxygen Species to Maintain Viral Gene Silencing during Latency
Source: Int J Mol Sci. 2022 Sep 20;23(19):11017. doi: 10.3390/ijms231911017 (PMC9569889; doi:10.3390/ijms231911017)
Supplement: Supplementary file 1 [file ijms-23-11017-s001.zip › ijms-1822639-supplementary.pdf]

Supplementary Figures

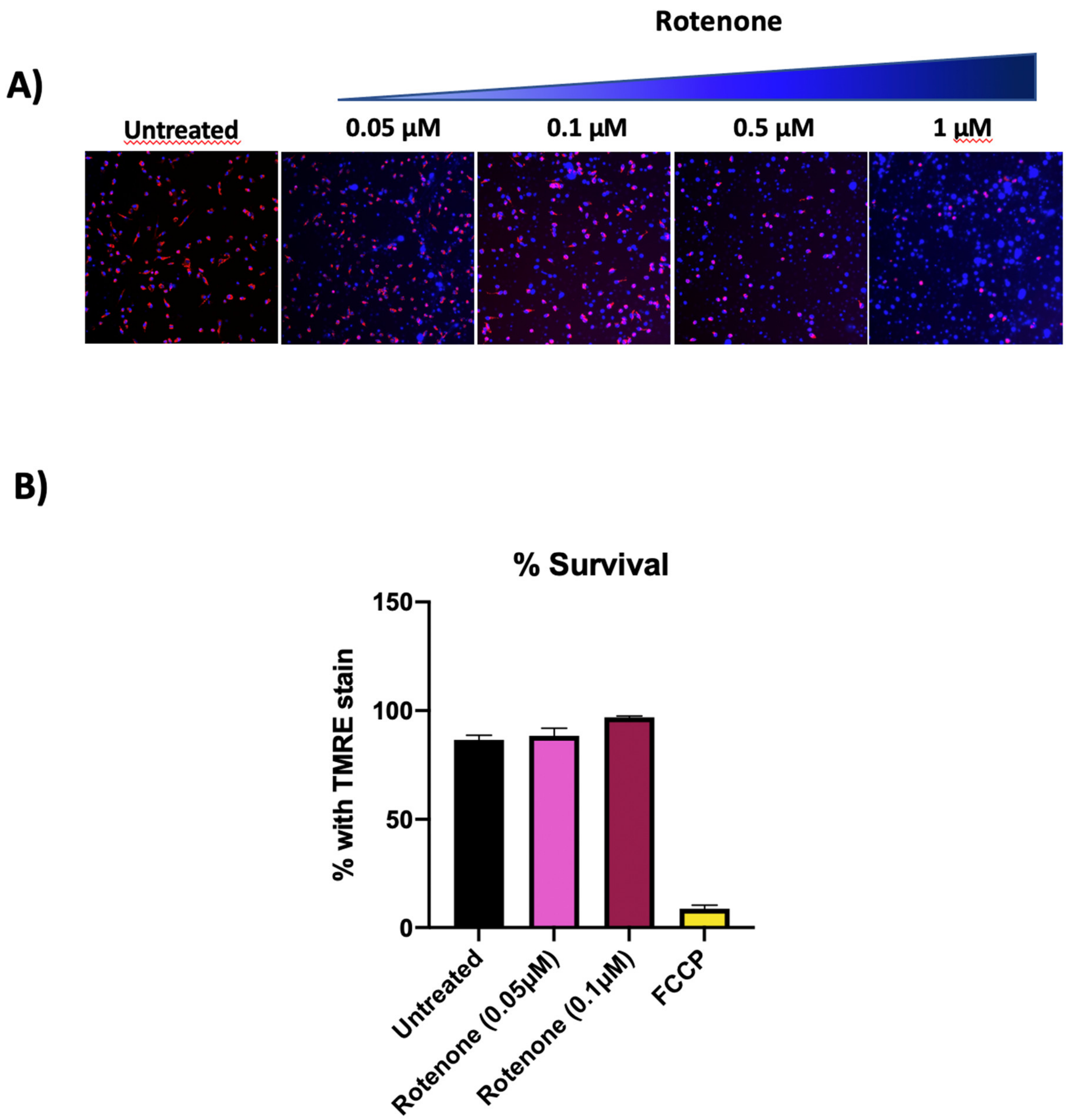

Figure S1. Low levels of rotenone cause negligible cell death in CD14+ monocytes. (A) CD14+ monocytes were treated with the indicated concentrations of rotenone. 5 days post treatment, cells were stained with TMRE (a stain for actively respiring mitochondria) and Hoechst and then imaged by fluorescence microscopy. (**B)** CD14+ monocytes were treated with low concentrations of rotenone for 5 days, or with FCCP FCCP (an uncoupler of mitochondrial oxidative phosphorylation) for 10mins. Cells were TMRE and Hoechst stained. The % of TMRE+ve cells was enumerated in 3 different fields of view for each condition indicated.

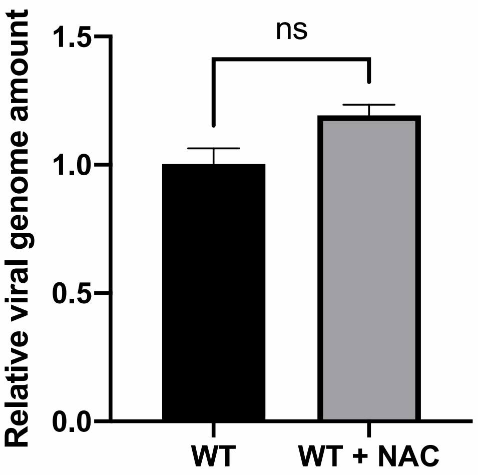

Figure S2. NAC does not affect viral entry. CD14+ monocytes were pretreated with or without 1mM NAC for 2 hrs, before being infected with WT SV40-GFP TB40E virus at an MOI of 5. 1 day post infection, DNA was harvested and subject to qPCR for genomic viral DNA and the housekeeping gene, GAPDH. Graph shows mean and standard error of experiments performed in triplicate.

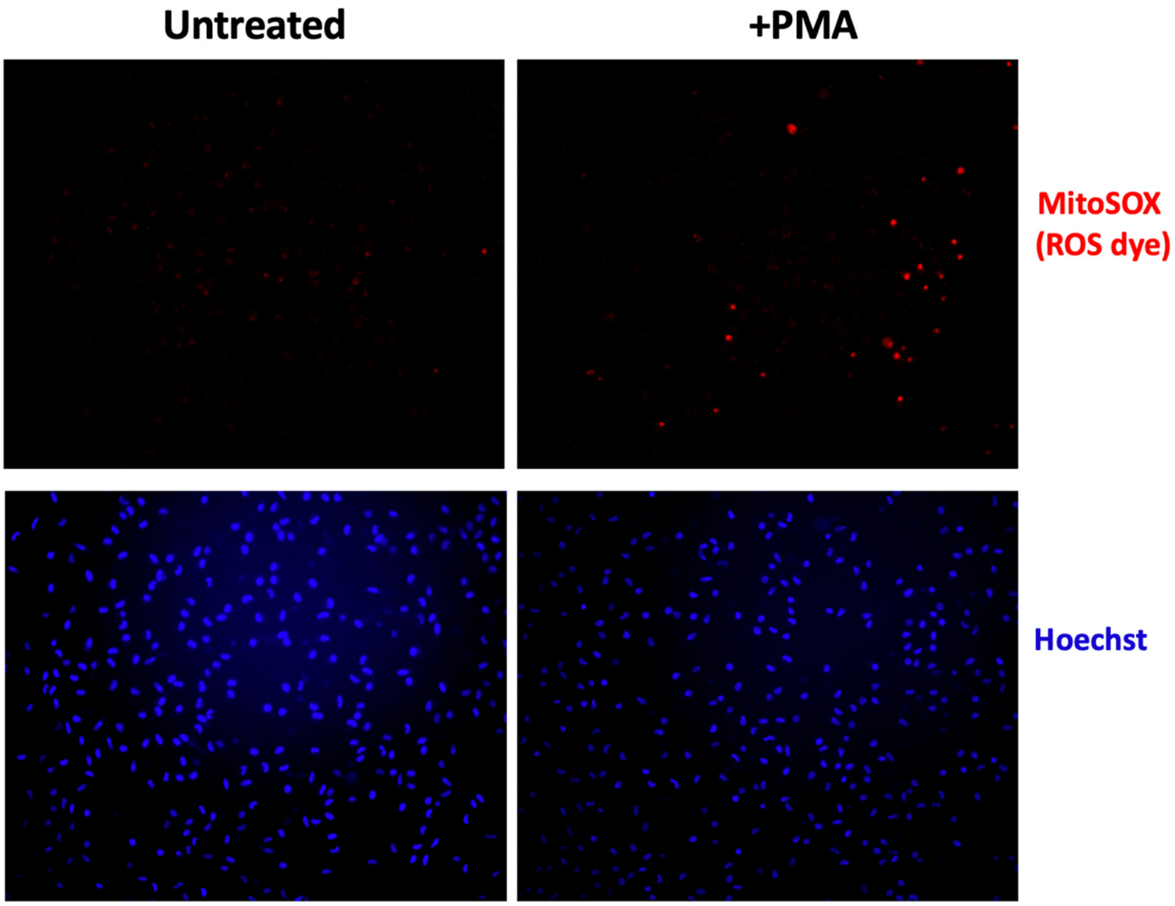

Figure S3. The myeloid differentiation inducing agent, PMA, stimulates ROS production in monocytes. CD14+ monocytes were isolated and treated with 50 ng/µl PMA for 4 hours. Cells were then stained with the superoxide dye, MitoSOX and Hoechst and imaged under a fluorescence microscope.

A)

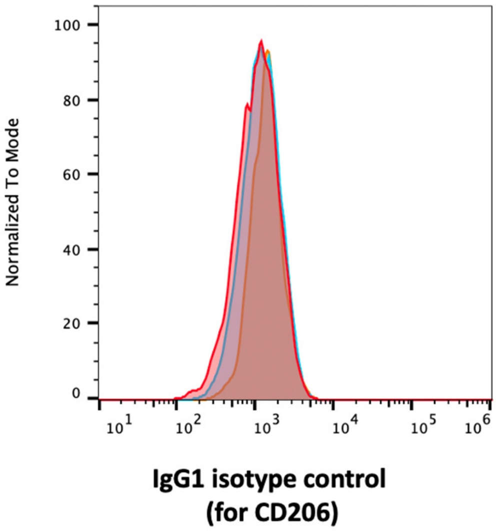

	Sample Name	Mean : FL4-H
(red)	Unstained	1220
(blue)	Untreated	1372
(orange)	+IL-4/GM-CSF + LPS	1495

B)

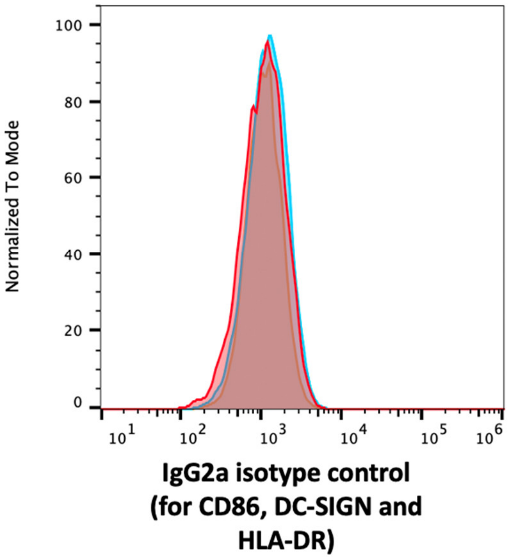

	Sample Name	Median : FL4-H
(red)	Unstained	1072
(blue)	Untreated	1192
(orange)	+IL-4/GM-CSF + LPS	1060

Figure S4. Isotype control antibody staining. CD14+ monocytes were untreated or differentiated and matured with IL-4/GM-CSF + LPS . Cells were stained with isotype control antibodies: (**A)** IgG1 for CD206, and (**B)** IgG2a for CD86, DC-SIGN and HLA-DR.
